# Usp5 links suppression of p53 and FAS levels in melanoma to the BRAF pathway

**DOI:** 10.18632/oncotarget.2140

**Published:** 2014-06-26

**Authors:** Harish Potu, Luke F. Peterson, Anupama Pal, Monique Verhaegen, Juxiang Cao, Moshe Talpaz, Nicholas J. Donato

**Affiliations:** ^1^ Department of Internal Medicine, University of Michigan Comprehensive Cancer Center, Ann Arbor, MI; ^2^ Department of Dermatology, University of Michigan Comprehensive Cancer Center, Ann Arbor, MI; ^3^ Department of Dermatology, Boston University School of Medicine, Boston, Massachusetts

**Keywords:** Usp5, ubiquitination, BRAF, p53, FAS, melanoma

## Abstract

Usp5 is a deubiquitinase (DUB) previously shown to regulate unanchored polyubiquitin (Ub) chains, p53 transcriptional activity and double-strand DNA repair. In BRAF mutant melanoma cells, Usp5 activity was suppressed by BRAF inhibitor (vemurafenib) in sensitive but not in acquired or intrinsically resistant cells. Usp5 knockdown overcame acquired vemurafenib resistance and sensitized BRAF and NRAS mutant melanoma cells to apoptosis initiated by MEK inhibitor, cytokines or DNA-damaging agents. Knockdown and overexpression studies demonstrated that Usp5 regulates p53 (and p73) levels and alters cell growth and cell cycle distribution associated with p21 induction. Usp5 also regulates the intrinsic apoptotic pathway by modulating p53-dependent FAS expression. A small molecule DUB inhibitor (EOAI3402143) phenocopied the FAS induction and apoptotic sensitization of Usp5 knockdown and fully blocked melanoma tumor growth in mice. Overall, our results demonstrate that BRAF activates Usp5 to suppress cell cycle checkpoint control and apoptosis by blocking p53 and FAS induction; all of which can be restored by small molecule-mediated Usp5 inhibition. These results suggest that Usp5 inhibition can provide an alternate approach in recovery of diminished p53 (or p73) function in melanoma and can add to the targeted therapies already used in the treatment of melanoma.

## INTRODUCTION

Melanoma is a very aggressive skin cancer characterized by several genetic defects, a high metastatic capacity and extraordinary resistance to chemotherapy [1]. However, recent success has been reported in melanoma patients using kinase-targeted therapy [2]. BRAF is a serine/threonine kinase that is mutated and constitutively activated in ~60% of melanoma patients [3]. BRAF kinase inhibitors (i.e. Vemurafenib) induce objective clinical responses in most mutant BRAF positive patients. However, clinical responses are typically short-lived and most patients relapse within 6 to 9 months of initial therapy [4]. Recent studies have used small molecule inhibitor arrays to define protein profiles that mediate desensitization of mutant BRAF melanoma cells to BRAF and other kinase inhibitors [5]. Such profiling demonstrates the capacity to define new drug combinations that could enhance melanoma responsiveness or delay resistance to vemurafenib or other kinase pathway inhibitors.

Signal transduction cascades can also be regulated by ubiquitination with the majority of signaling pathways requiring both phosphorylation and ubiquitination for full regulatory control [6-10]. Ubiquitination alters the structure, localization, destruction and function of enzymes or proteins and emerging evidence suggest that defects or unbalanced regulation of this process plays an important role in multiple diseases, including cancer [11]. Ub removal is catalyzed by a relatively small number of enzymes (<100) with deubiquitinase (DUB) activity [12]. Knockdown studies suggest that DUBs may be valid therapeutic targets [12-16]. However, only a few specific DUB inhibitors have been described and none have entered clinical studies [17-19]. Very little is known regarding the role of DUBs in melanoma biology or therapy. In this study, we used a combination of approaches to define a role for deubiquitinases (DUBs) in melanoma cell signaling and survival. We first noted a change in ubiquitinylated protein content and unanchored Ub chains in BRAF mutant cells treated with vemurafenib which was mediated through inhibition of Usp5. We used Usp5 knockdown (KD), overexpression and enzyme inhibition to demonstrate that Usp5 suppresses p53 and FAS levels in melanoma and is associated with loss of checkpoint control and apoptotic sensitivity to kinase inhibitors and other agents. We show that p53 and FAS levels could be restored by a small molecule DUB inhibitor and DUB inhibition completely suppressed melanoma tumor growth *in vivo* without overt toxicity. These results highlight an unexpected link between aberrant kinase signaling and the ubiquitin-proteosome pathway through activation of a deubiquitinase capable of regulating multiple downstream effectors. It also supports the potential for DUB inhibitors to improve or sustain kinase-inhibitor anti-tumor activity.

## RESULTS

### Modulation of ubiquitin content and DUB activity in BRAF mutant melanoma

**Figure 1:**
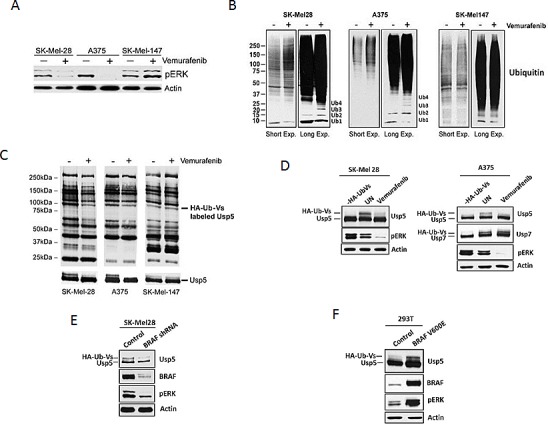
BRAF regulates Usp5 activity A. BRAF mutant (SK-Mel28, A375) and non-mutant (SK-Mel147) melanoma cells were treated with 5 μM vemurafenib for the 24 hr before cell lysates were subjected to immunoblotting for the protein indicated. B. BRAF mutant and non-mutant cells were treated with DMSO (-) or 5 μM vemurafenib (+) for 24 h before cell lysates were subjected to immunoblotting for total ubiquitin. The mobility of mono-, di-, tri- and tetra-Ub is denoted. C. Melanoma cells were incubated with 5 μM vemurafenib for 24 hr before DUB activity was assessed in lysates by HA-UbVS labeling followed by HA blotting (top). Migration of HA-Ub-Vs labeled Usp5 is denoted (top). Immunoblotting of the same membrane for Usp5 is shown at the bottom. D. Left – SK-Mel28 cells were incubated with or without vemurafenib for 24 hours before lysates were labeled by incubation with HA-UbVS. Usp5 was immunoblotted as a measure of its activation. Activated Usp5 appears as doublet above the Usp5 band. pERK and actin immunoblots are also shown. Right – Similar analysis was conducted with A375 cells. Usp7 was immunoblotted as a control. E. Control or BRAF KD SK-Mel28 cells were assessed for BRAF, pERK and Usp5-specific DUB activity by HA-UbVS labeling followed by Usp5 blotting. F. HEK293T cells transfected with control or BRAF^V600E^ expression vector were assessed for BRAF and pERK by immunoblotting. Usp5-specific DUB activity was assessed as described in D.

We confirmed differential vemurafenib activity in BRAF mutant (A375, SK-Mel-28) and non-mutant (SK-Mel-147) melanoma cell lines with regard to growth and pERK inhibition occurring only in BRAF mutant cells (Fig 1A and Supplemental Fig. 1A). We assessed total protein ubiquitination in vemurafenib treated and control cells and noted that pERK inhibition was associated with an increase in total protein ubiquitination (Fig 1B). Long-term exposures demonstrated that monomeric Ub was diminished while Ub polymers (Ub_2-4_) were increased, consistent with previous reports of increased Ub polymers in DUB inhibited or knockdown cells [20]. To determine whether DUB activity was affected by vemurafenib, melanoma cell lysates derived from control and treated cells were subjected to DUB activity assessment using an irreversible DUB inhibitor that covalently modifies active DUBs with HA-Ub. DUB activity was assessed by HA blotting (Fig. 1C) and confirmed by monitoring a DUBs mobility shift due to its covalent modification with HA-Ub (Fig. 1C,D,E) [21, 22]. DUB inhibition was detected in vemurafenib-responsive (SK-Mel28 and A375) cells and we noted a consistent change in a DUB (100kDa) identified as Usp5 by LC/MS/MS of the excised protein band (data not shown) and direct immunoblotting (Fig. 1C,D). Vemurafenib did not alter Usp7 activity, a 130kDa DUB previously shown to regulate p53 turnover. DUB activity was also compared in control and BRAF KD cells. BRAF shRNA reduced pERK levels and Usp5 activity (Fig.1E). To confirm DUB regulation through BRAF activation, mutant BRAF (V600E) was expressed in HEK293T cells and DUB activity assessments were used to demonstrate increased Usp5 activity in cells expressing BRAF^V600E^ (Fig. 1F). These results confirm that BRAF mutation or activation results in changes in the activity of specific DUBs, including Usp5.

### Usp5 regulates melanoma cell growth

**Figure 2:**
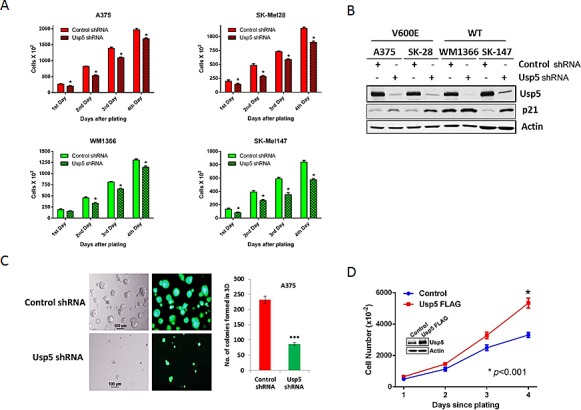
Usp5 regulates melanoma cell growth A. BRAF mutant (red) and non-mutant (green) control and Usp5 KD melanoma cells were plated at 5,000 cells per plate (Day 0) and cell counts were conducted daily. Each value represents the average +/- S.D. of 3 independent counts. The p value for each grouping (control vs. Usp5) was <0.05 (*). B. Control or Usp5 KD melanoma cells (as noted) were immunoblotted for Usp5, p21 and actin. C. Phase contrast and eGFP fluorescent images of A375 cells with stable Usp5 knockdown (KD) and control cells grown on matrigel for 7 days. The bar graph shows the number of single round acinar structures +/- S.D. obtained by counting 100 structures from 3 separate wells. D. Control or FLAG-Usp5 A375 cells (inset), were plated at 5,000 cells per culture dish and counted daily for 4 days. Each value represents the average +/- S.D. of 3 independent counts. * *p<0.05*.

Two mutant and two non-mutant BRAF melanoma cell lines were subjected to Usp5 KD and their growth kinetics were assessed over four days after plating equal numbers of initiating cells. As shown in figure 2A, Usp5 KD reduced the rate of growth of both BRAF mutant and non-mutant cells. Cell cycle analysis demonstrated that Usp5 is important for entry into G2/M (Supplemental Fig. 1B). Growth inhibition was associated with induction of p21 in Usp5 KD cells (Fig. 2B) and Usp5 KD caused >3-fold reduction in both the number and size of A375 colonies when plated on Matrigel, which partially replicates an *in vivo* 3D growth environment (Fig. 2C). Overexpression of Usp5 nearly doubled the rate of melanoma growth when compared to control cells (Fig. 2D).

### Usp5 regulates apoptotic responsiveness to kinase inhibition

**Figure 3:**
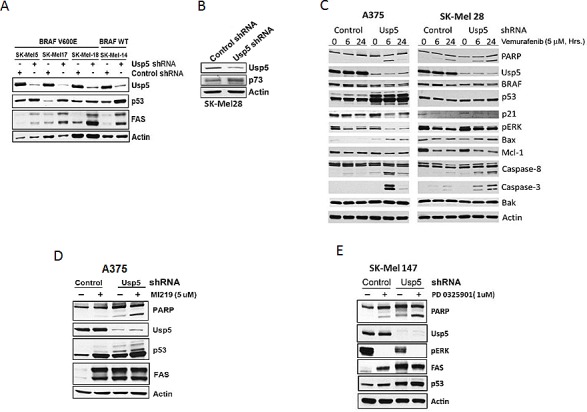
Usp5 regulates apoptotic responsiveness to kinase inhibition A. Control and Usp5 KD melanoma cells with wild-type p53 and mutant or non-mutant BRAF expression (as indicated) were immunoblotted for Usp5, p53, FAS and actin. B. Control and Usp5 KD SK-Mel28 cells were immunoblotted for Usp5, p73 and actin. C. Control or Usp5 KD SK-Mel28 and A375 cells were treated with 5 μM vemurafenib for the interval noted before cell lysates were immunoblotted for the protein indicated. D. Control and Usp5 KD A375 cells were treated with 5 μM MI219 for 24 hr before cell lysates were immunoblotted for the protein indicated. E. Control and Usp5 KD SK-Mel 147 cells were treated with or without 1 μM PD 0325901 for 6 hr. Cell lysates were subjected to immunoblotting for the protein indicated.

To determine whether BRAF mediated-DUB activation regulates the cellular response to vemurafenib, control and Usp5 KD cells were treated with vemurafenib for the interval indicated. Usp5 KD resulted in morphologic changes in A375 cells (Supplemental Fig. 1C) and >3-fold increased apoptotic responsiveness (annexin positivity) to vemurafenib (Supplemental Fig. 1D) in BRAF mutant cell lines. Usp5 was previously shown to regulate p53 entry into and destruction by the 20S proteasome [20]. Usp5 KD resulted in increased levels of p53 protein and FAS in a panel of melanoma cells (Fig. 3A). Usp5 KD resulted in up-regulation of p53 in w/t p53 A375 cells and up-regulation of p73 in p53 mutant SK-Mel28 cells (Fig. 3B), suggesting that both proteins can be modulated by Usp5. In both w/t and mutant p53 expressing cells, Usp5 KD enhanced the onset or extent of apoptosis induced by vemurafenib, with evidence for activation of both the intrinsic and extrinsic pathway (Fig. 3C).

To determine whether Usp5 also regulates apoptotic responsiveness to other stimuli, Usp5 KD and control cells were treated with MI219, an HDM2 inhibitor that prevents p53 ubiquitination. MI219 increased p53 protein and FAS levels but had limited impact on PARP cleavage (Fig. 3D). In Usp5 KD cells, p53 levels and PARP cleavage were further enhanced by MI219. Similar results were obtained in non-mutant BRAF cells treated with a MEK inhibitor (Fig. 3D).

### Usp5 regulates p53 and FAS to affect kinase-inhibitor induced apoptosis

**Figure 4:**
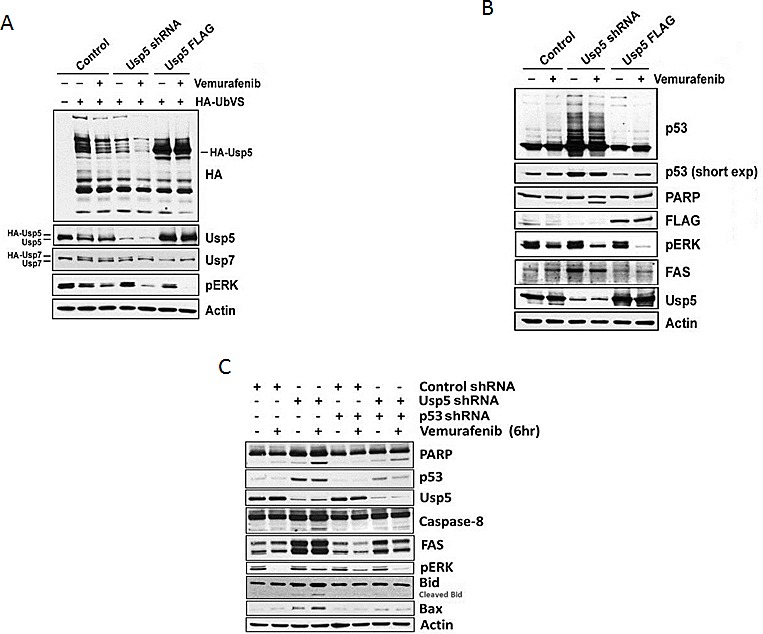
Usp5 regulates p53-mediated cell death A. Control, Usp5 KD or Usp5-FLAG A375 cells were left untreated or treated with 5 μM vemurafenib for 6 hr before assessing Usp5, Usp7 and pERK expression (bottom) by blotting. DUB activity was assessed as in figure 1C. B. A375 cell lysates (from Fig. 4A) were blotted for the protein indicated. Long and short film exposures for p53 blots are shown. C. Control, Usp5, p53 single and dual KD A375 cells were treated with or without vemurafenib for 6 hr before cell lysates were subjected to immunoblotting for the protein indicated.

To confirm modulation of vemurafenib activity by Usp5, A375 control, Usp5 KD (Usp5 shRNA) and Usp5 overexpressing (Usp5 FLAG) cells were left untreated or treated with vemurafenib before examining Usp5 expression, activity, p53 protein levels and apoptosis. Usp5 KD and over-expression altered Usp5 DUB activity and its vemurafenib-mediated inhibition (Fig. 4A). Usp5 KD consistently led to p53 induction and accumulation of ubiquitinated p53 adducts (Fig. 4B, center lanes) while Usp5 overexpression diminished p53 content. Vemurafenib did not alter Usp7 activity, which also regulates p53 levels in some cells [19]. Increased p53 levels in Usp5 KD cells were associated with FAS induction and the rapid onset of apoptosis upon vemurafenib treatment (Figs. 3 and 4). To assess the role of p53 induction in apoptosis and FAS regulation in cells with altered Usp5 expression, we compared apoptotic activity in cells with either Usp5 knockdown or dual knockdown of Usp5 and p53. Usp5 KD resulted in increased p53, FAS and Bax protein expression as well as increased Bid and PARP cleavage in response to vemurafenib (Fig. 4C). In dual Usp5/p53 KD cells, these activities were blocked, suggesting a prominent role for both Usp5 and p53 in the activation of vemurafenib-mediated cell death.

### Usp5 regulates FAS levels and contributes to apoptotic sensitivity in melanoma

**Figure 5:**
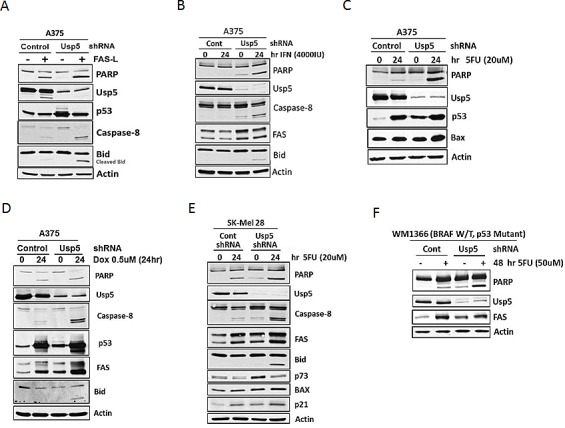
Usp5 regulates apoptotic sensitivity in melanoma A. Control and Usp5 KD A375 cells were left untreated or treated with 250 ng/mL FAS-L for 6 hrs before cell lysates were subjected to immunoblotting for the protein indicated. B. A375 cells (from Fig. 5A) were left untreated or treated with IFN-α (4000 IU) for 24 hr before immunoblotting for the protein indicated. C. A375 cells (from Fig. 5A) were left untreated or treated with 5FU for 24 hr before lysates were immunoblotted for the protein indicated. D. A375 cells (from Fig. 5A) were left untreated or treated with doxorubicin for 24 hr before cell lysates were immunoblotted for the protein indicated. E. Control and Usp5 KD SK-Mel28 cells were left untreated or treated with 5FU for 24 hr before lysates were subjected to immunoblotting for the protein indicated. F. Control and Usp5 KD WM1366 cells were left untreated or treated with 5FU for 48 hr. Cell lysates were immunoblotted for the protein indicated.

To confirm a role for Usp5 in FAS induction and function, control and Usp5 KD cells were treated with FAS-L and activation of the extrinsic apoptotic pathway was assessed. FAS-L resulted in limited activation of caspase 8, Bid and PARP cleavage, which was highly amplified by Usp5 KD (Fig. 5A). Similar results were obtained in cells treated with IFN-α, a FAS-inducing apoptotic cytokine [23, 24] used in the clinical treatment of melanoma [25] (Fig. 5B). BRAF inhibition should release apoptotic suppression through reduced Usp5 activity, increased FAS expression and engagement of apoptosis, through the extrinsic caspase cascade. To test that potential, cells were treated with vemurafenib for extended intervals and assessed for FAS and Bax induction, caspase 8 activation, Bid and PARP cleavage. Vemurafenib treatment led to an early increase in protein ubiquitination, FAS and Bax induction (24 hours), followed by caspase 8, Bid and PARP cleavage after 48-72 hours (supplemental fig. 2). Vemurafenib reduced DR5 levels in SK-Mel19 cells, in agreement with previous studies [26](Supplemental Fig. 3A). BRAF^V600E^ expression in HEK293T cells resulted in an increase in DR4 and DR5, but a reduction of FAS and p53 levels (Supplemental Fig. 3B). FAS reduction by Usp5 appears to be mediated at the transcriptional level, possibly through down-regulation of p53 and other factors (Supplemental Fig. 3C).

Since Usp5 was recently reported to play a role in DNA damage repair [27], we assessed the effect of Usp5 KD on 5FU and Doxorubicin apoptotic responsiveness. Usp5 KD enhanced caspase activation, primarily through increased caspase 8 activation in both p53 wild-type and mutant cells (Fig. 5C-F). As noted in figure 3C, Usp5 also regulates p73 (Fig. 5E) and may play a role in the apoptotic responsiveness of p53 mutant tumors [28].

### Usp5 depletion overcomes acquired resistance to vemurafenib in melanoma

**Figure 6:**
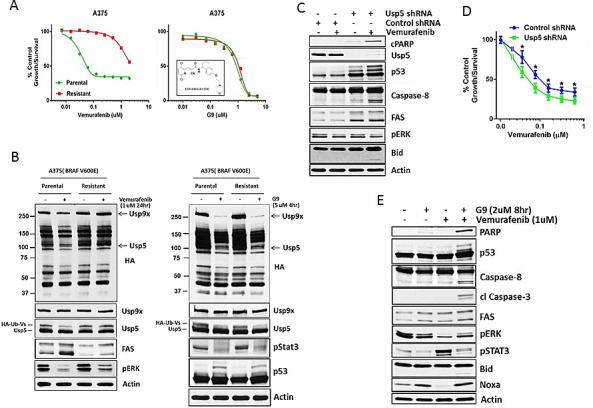
Usp5 depletion or inhibition overcomes acquired resistance to vemurafenib in melanoma A. A375 parental and resistant cells were treated with vemurafenib (left) or G9 (right; structure shown in inset) at the indicated concentration for 72 hr before cell growth was assessed by MTT assay. The results represent the average +/- S.D. of triplicate assays. B. Left- A375 parental and resistant cells were treated with vemurafenib (24 hr) as indicated before cell lysates were assessed for DUB activity (as in Fig. 1C; top panel) or the protein indicated (bottom panel). Usp5 and Usp9x DUB activity are denoted (arrows). Right - A375 parental and resistant cells were treated with G9 (4 hr) as indicated before cell lysates were assessed for DUB activity (top panel) or the protein indicated (bottom panel). C. Control or Usp5 KD A375R cells were treated with vemurafenib (1 μM) for 24 hr before cell lysates were examined for the protein indicated. D. Control and Usp5 KD A375 cells were treated with the vemurafenib concentration indicated for 48 hr before cell growth was assessed by MTT assay. The results represent the average +/- S.D. of triplicate assays. * p<0.05. E. A375R cells were treated with vemurafenib or G9 alone or in combination before cell lysates were examined for the protein indicated.

To assess potential clinical relevance of Usp5 activity in melanoma, isogenic vemurafenib sensitive and resistant A375 melanoma cells (Fig. 6A) were treated with a novel, small molecule DUB inhibitor. We conducted extensive structure-activity relationship studies on WP1130, a Usp5/Usp9x/Usp14/UCH-L1/UCH-L5 inhibitor [22, 29], to improve its safety, solubility and anti-tumor activity in mice. The compound EOAI3402143 (or G9; Inset - Fig. 6A) retained potent Usp9x and Usp5 inhibitory activity (Supplemental Fig. 4A). We assessed its effects on vemurafenib sensitive and resistant cells and noted similar *in vitro* anti-tumor efficacy (Fig. 6A; IC_50_ 1 μM). We compared DUB activity in vemurafenib and G9 treated cells and show that vemurafenib suppressed Usp5 activity in sensitive but not resistance cells, although pERK was reduced by kinase inhibitor in either cell type. Vemurafenib also failed to induce FAS in resistant cells (Fig. 6B - left). G9 reduced Usp5 (and Usp9x) activity in both cell types (Fig. 6B), increased p53 levels and retained pStat3 inhibitory activity as previously described for the WP1130 compound [22, 30, 31]. To determine whether Usp5 KD (or G9) could overcome vemurafenib resistance, Usp5 KD A375R cells were left untreated or treated with vemurafenib (for 24 hrs) before assessing caspase activation, PARP and Bid cleavage. Usp5 KD enhanced p53 accumulation, increased FAS levels and activated apoptosis in response to vemurafenib (Fig. 6C and E). Similar results were obtained in A375R Usp5 KD cells treated with a MEK inhibitor (Supplemental Fig. 4C). In addition, Usp5 KD reduced the vemurafenib IC50 concentration in A375 cells by ~2-fold (Fig. 6D). In A375R cells, G9 reduced pERK, pStat3 and elevated NOXA levels, the latter related to Usp9x inhibition by G9 (Fig. 6E). When combined with vemurafenib or 5FU (Supplemental Fig. 4D), G9 induced PARP and Bid cleavage with activation of caspases 8 and 3.

### DUB inhibition suppresses melanoma growth *in vivo*

**Figure 7:**
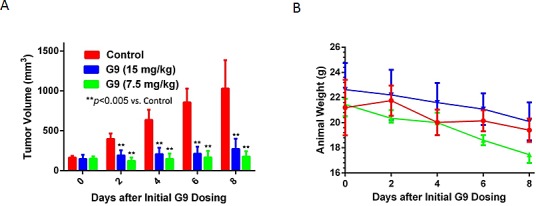
G9 suppresses melanoma growth *in vivo* A. NSG mice were subcutaneously inoculated with A375 cells. Animals with established tumors were size matched and allocated to four per treatment group (vehicle, G9). The first day of treatment was day 12 post tumor inoculation. Treatment was with G9 (15, 7.5 mg/kg) in DMSO: PEG (1:1) every day by i.p. B, NSG mouse weight measurements recorded throughout the treatment interval.

A375 tumors grown as subcutaneous implants in NSG mice were separated into three groups and received once daily ip injections with vehicle control (PEG300/DMSO) or G9 at doses of 7.5 or 15 mg/kg. Tumor growth, animal weight, behavior and mobility were monitored during treatment. As shown in figure 7A, both 7.5 and 15 mg/kg dosing completely suppressed tumor growth, with control mice reaching maximal tumor burden by day 8 of treatment. Cessation of G9 resulted in tumor growth which approached control levels 10 days after stopping G9 injection (data not shown). Weight loss was not significantly different between control and G9 treated mice and we did not observe changes in behavior or mobility in control or G9 treated mice (Fig. 7B). These results suggest that G9 is well tolerated and effective as mono-therapy for melanoma.

## DISCUSSION

Understanding of the events activated by mutant BRAF and the cellular consequences of its inhibition may provide insight into a means of prolonging or deepening the therapeutic response. Toward that goal, we assessed the role of ubiquitin modulation in the BRAF cascade and response to its inhibition. Usp5 was consistently suppressed, but not fully inhibited, by kinase inhibitor or BRAF knockdown. Expression of BRAF^V600E^ in BRAF mutant naïve cells resulted in increased expression and activation of Usp5, suggesting that pathway activation or phospho-regulation underlie Usp5 activation. However, we have been unable to detect changes in Usp5 phosphorylation in vemurafenib-treated melanoma or BRAF^V600E^ over-expressing cells, suggesting other mechanisms of activation, (i.e. allosteric) may be operant [32, 33].

Usp5 regulates both cell cycle progression and apoptotic responsiveness to vemurafenib and other targeted therapies, at least partially associated with p53 and p73 induction, as vemurafenib apoptotic activity was increased even in p53 mutant cells (Fig. 3A, 5E, 5F). Previous studies of p53 regulation by Usp5 demonstrated p53 stabilization through accumulation of unanchored poly-ubiquitin chains which compete with p53 (but not HDM2) for entry into the proteasome [20]. This may also play a role in recruitment of Usp5 into the DNA damage response as recently described [27]. Of note, the effect of Usp5 KD on p53 is distinct from that of an HDM2 inhibitor (MI219), which blocks p53 ubiquitination to circumvent its destruction [34]. Both Usp5 KD and MI219 alone lead to p53 increase and FAS induction, which were further elevated by co-treatment. This suggests that Usp5 and HDM2 regulate p53 levels by distinct mechanisms. Usp5 inhibition could provide an alternate approach in reactivation of p53 (and p73), which are rarely mutated, but functionally inactivated in melanoma [35, 36].

These studies also uncover links between mutant BRAF expression and suppression of FAS in melanoma. We noted caspase 8 activation and PARP cleavage after long-term BRAF inhibition, preceded by up-regulation of FAS, but down-regulation of DR5, in agreement with other studies of DR5 regulation by activated RAS/RAF [26]. We noted suppression of FAS levels by expression of activated BRAF, which was relieved following treatment with vemurafenib. Thus, extrinsic caspase activation is suppressed by mutant BRAF activity, at least in part through BRAF regulation of Usp5 activity and p53 induction. However, loss of Usp5 regulation by BRAF inhibitor in vemurafenib-resistant A375R may underlie clinical resistance to BRAF inhibitors in some patients, but additional studies will be needed to make that determination. Usp5 KD improved apoptotic activity in both mutant and non-mutant BRAF melanoma, suggesting that Usp5 inhibitors would be useful in a broader patient population, including those that are candidates for other therapies [MEK inhibitor, immune-regulators (ipilimumab, IFN-α) DNA-damaging chemo-therapies].

While the safety and efficacy of Usp5 inhibition has not yet been defined, there is potential for targeting this or other DUBs for therapeutic purposes. This approach has not yet been tested, primarily due to the limited number of Usp5 inhibitors available. The DUB inhibitor described here reduced Usp9x and Usp5 activity, induced p53, FAS and NOXA, reduced pStat3 levels and amplified vemurafenib apoptotic activity *in vitro*. G9 was well tolerated in mice and it effectively blocked melanoma growth, even at low doses. Even lower G9 doses may be effective in melanoma tumors, possibly due to its capacity to affect several pathways essential for melanoma growth. As noted with other DUB inhibitors [18, 37], broad target inhibition may be more effective anti-tumor agents than more specific inhibitors. The clinical efficacy and safety of this approach will need to be carefully examined.

Overall, this study demonstrates that aberrant kinase activity can modify the ubiquitin-proteasome pathway through modulation of DUBs, as we previously described in Bcr-Abl expressing cells [38]. Since the cellular activities and substrates controlled by DUBs are now starting to emerge, appropriate use of DUB inhibitors alone or in combination with other pathway or kinase inhibitors may provide a novel therapeutic approach in melanoma and other cancers.

## MATERIALS AND METHODS

### Cell Lines

A375, SK-Mel28, SK-Mel19, SK-Mel5, SK-Mel14 SK-Mel18, SK-Mel17, SK-Mel147, WM1366 and HEK293T cells were maintained in Dulbecco's Modified Eagle's Medium (DMEM) with 10% heat-inactivated FBS (Atlanta Biological), 2 mM L-glutamine and 1% penicillin/streptomycin (GIBCO).

### Chemical Reagents

EOAI3402143 (referred to as G9) was synthesized and provided by Evotec (UK), (Abingdon Oxfordshire, UK). Other reagents used in this study were obtained from the following sources: hemagglutinin-tagged ubiquitin vinyl methyl sulfone (HA-UbVS; Boston Biochem); Vemurafenib (PLX4032; Chemie Tek); PD0325901 (Cayman Chemical); MI-219 (A kind gift of Dr. Shaomeng Wang, University of Michigan). All reagents were made up and stored frozen as 10 mM stock solutions.

### shRNA-mediated gene silencing

Melanoma cells were infected with the lentiviral expression system for short hairpin RNA (shRNA)-mediated BRAF and Usp5 knockdown and their control; pGIPZ Control, pGIPZ-USP5, and pGIPZ-BRAF were obtained from Open Biosystems. Knockdown of p53 was achieved with the following sense targeting sequence: p53; 5′-GACTCCAGTGGTAATCTAC-3′ cloned into pRetrosuperpuro (Oligoengine, Seattle, WA, USA). pBabe-puro-BRAF-V600E and pDEST-LTR-N-FLAG-HA-IRES-USP5 expression vectors was obtained from Addgene. HEK293T cells were transfected with the lentiviral packaging vectors pMD2.G and psPax2 (Addgene) together with the shRNA vectors to produce virus using PolyFect as described by the manufacturer (QIAGEN). The medium was changed to DMEM with 10% fetal bovine serum and after 48 hours, and the viral supernatant was collected. Viral supernatant containing 4 μg/mL of Polybrene (Sigma-Aldrich) was added to each melanoma cell line. After puromycin selection, Usp5 stable knockdown, overexpressing or control cells were used for analysis.

### Three-dimensional cultures (3D)

Equal numbers of viable control and knockdown cells from each cell type (500-2000 cell/well) were grown on growth factor reduced matrigel (Catalog # 354230; BD transduction) for 5 – 7 days following previously described protocols [39]. Images were recorded in both phase-contrast and fluorescent mode on a Leica inverted microscope. The percentage of single round acinar structures was calculated by counting at least 100 structures from 3 wells of an 8-well chamber slide containing control and knockdown cells.

### DUB-labeling assays

To assay DUB activity, melanoma cells were lysed in DUB buffer (50 mM Tris pH 7.2, 5 mM MgCl_2_, 250 mM sucrose, protease inhibitor cocktail (Roche), 1 mM NaF and 1 mM PMSF) for 10 minutes at 4°C, followed by brief sonication. The lysates were centrifuged at 20,000*g* for 10 minutes, and the supernatant was used for DUB labeling. Equal amounts of lysate (20 μg) were incubated with 2 μM of HA-UbVS for 75 min at 37°C, followed by boiling in reducing sample buffer and resolving by SDS-PAGE. HA immunoblotting was used to detect DUB labeling as previously described [22].

### Lysate preparation and Western blotting

Total cell lysates were prepared by sonicating and boiling cell pellets in 1X Laemmli reducing sample buffer. Detergent-soluble cell lysates were prepared by lysing cells in cold isotonic lysis buffer (10 mM Tris-HCl, pH 7.5, 0.1% Triton X-100, 150 mM NaCl, along with protease inhibitor cocktail and 1 mM PMSF for 15 minutes on ice and centrifuged for 10 minutes at 20,000 *g*. The clarified supernatant was used as the detergent soluble cell fraction. Lysates were electrophoresed (SDS-PAGE gels) and transferred to nitrocellulose membranes (Whatmann). Proteins were detected by immunoblotting.

Antibodies used in this study were purchased from the following sources: anti-actin and FLAG (Sigma-Aldrich); anti-ubiquitin clone P4D1, goat, anti-rabbit/mouse/rat IgG-conjugated horseradish peroxidase, p21, ERK and Mcl-1 (Santa Cruz Biotechnology); USP7, USP5, BRAF and p73 (Bethyl Laboratories); anti–poly(ADP-ribose) polymerase (PARP), pERK, Caspase8, Caspase3, Bid, Bax, Bak (Cell Signaling Technology); anti-HA (clone 3F10; Roche Applied Science); CD95/Fas (Clone EPR5700; Epitomics); anti-NOXA, p53 (DO-1; CalBiochem); anti-DR4, DR5 (ProSci incorporated) and FAS monoclonal antibody (CH11; Millipore).

### Cell proliferation, viability and apoptosis assays

Cell proliferation was assessed by MTT staining as previously described [40]. Propidium iodide staining was used to measure apoptosis in control and treated cells. Briefly, 10^5^ cells/mL were harvested from tissue culture plates and centrifuged at 2,500*g* for 5 minutes at room temperature. Cell pellets were washed in PBS and re-suspended in 0.4 mL of cold propidium iodide. Samples were incubated at room temperature for 30 minutes in the dark, and analyzed by flow cytometry (Becton Dickinson). Annexin V-fluorescein isothiocyanate (FITC) staining was used to measure apoptosis. Cells were seeded in six-well plates and exposed to inhibitors for the interval indicated. The cells were then collected by trypsinization, washed with cold PBS, and stained with Annexin V-FITC for 10 min on ice. Positive cells were detected with flow cytometry.

### Reverse transcription-polymerase chain reaction (RT-PCR)

For quantitative analysis of gene expression by real-time PCR, total RNA was isolated from cell culture lysates according to the RNeasy Mini Kit protocol (Qiagen, Hilden, Germany). The purified RNA was reverse-transcribed by TaqMan Reverse Transcription reagent (Applied Biosystems, Carlsbad, CA) according to the manufacturer's protocol. The real-time PCR reactions were performed using iQ™ SYBR Green PCR Master Mix (Bio-Rad). For each sample, mRNA expression was normalized to the control value that was measured for glyceraldehyde 3-phosphate dehydrogenase (GAPDH) and reported as relative expression levels between samples.

### Xenograft studies

NSG [NOD/SCID/IL2r-g (null)] mice were injected mid-dorsally with 5 × 10^6^ A375 cells in 0.1 ml of Matrigel/DMEM suspension as previously described [29]. Tumors were allowed to establish to about 100 mm^3^, after which mice were tumor size matched and allocated to four per treatment group (vehicle or G9). G9 [in DMSO:PEG300 (1:1)] was administered by ip injection every day at either 15 or 7.5 mg/kg. Tumor volume was determined by caliper measurements (every other day) calculated with the following formula: volume = width (2) × length × height/2.

### Statistical analysis

Data points are shown as the mean ± SD. Student's t test was used to assess statistical performance using GraphPad Prism 6 and GraphPad InStat3.

## SUPPLEMENTARY MATERIAL AND FIGURES


