# Supplementary material for: Usp5 links suppression of p53 and FAS levels in melanoma to the BRAF pathway
Source: Oncotarget. 2014 Jun 26;5(14):5559–69. doi: 10.18632/oncotarget.2140 (PMC4170643; doi:10.18632/oncotarget.2140)
Supplement: Supplementary file 1 [file oncotarget-05-5559-s001.pdf]

Usp5 Links Suppression of p53 and FAS Levels in Melanoma to the BRAF Pathway

Supplemental Material

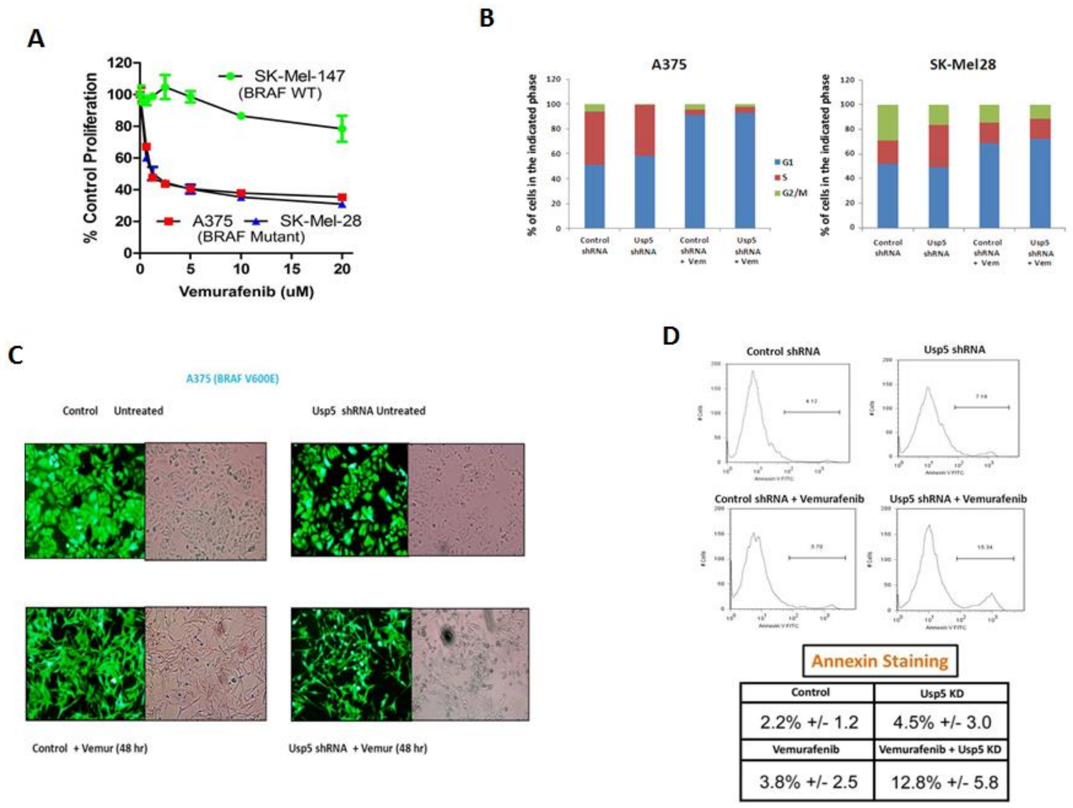

Figure S1: Usp5 regulates melanoma cell cycle progression and blocks cells in G1/S Phase. A. Melanoma cells were incubated with the indicated concentration of vemurafenib for 72 hr before cell growth was assessed by MTT assay (22). **B.** Melanoma cells expressing Usp5 or control shRNA were treated with or without 5 µM vemurafenib for 48 hr before cells were stained and analyzed for cell cycle distribution. **C.** A375 cells expressing Usp5 or control shRNA were treated with 5 µM vemurafenib for 48 hr. Cell images were acquired using an Olympus FluoView 500 confocal microscope using a 60× water immersion lens and 2× digital zoom (× 120 total magnifications). Images on left were acquired by fluorescence detection of eGFP in transfectants. Phase-contrast images on the right represent the same field as those used for eGFP detection (Kapuria et al., 2010). **D.** Top – A375 melanoma cells expressing Usp5 or control shRNA were treated with or without 5 µM vemurafenib for 24 hr before cells were analyzed by flow after Annexin-V/Propidium Iodide staining. Bottom – Analysis of annexin positivity in control and Usp5 KD A375 and SK-Mel28 cells left untreated or treated with vemurafenib (as described above). The values represent the ave. +/- S.D. from both cell lines in two independent experiments.

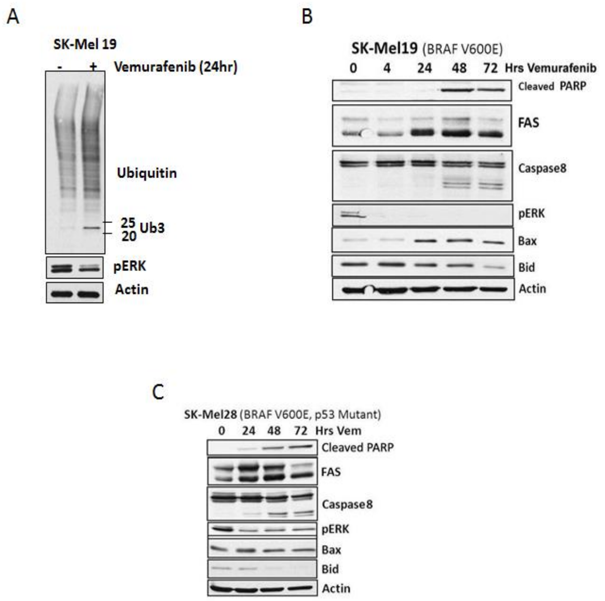

Figure S2:A. SK-Mel19 (BRAF^{V600E}) cells were treated with DMSO (-) or 5 μM vemurafenib (+) for 24 hr before whole cell lysates were resolved on high percent cross-linked gels and subjected to immunoblotting for total ubiquitin. The mobility of tri-Ub (Ub3) is denoted. pERK and actin were blotted as markers of BRAF inhibition and equal protein loading, respectively. **B.** SK-Mel19 cells were treated vemurafenib for the interval noted before measuring PARP cleavage, FAS, caspase 8 activation, pERK, Bax, Bid and actin. **C.** SK-Mel28 cells were treated with 5 μM vemurafenib for the interval noted. Cell lysates were subjected to immunoblotting for the protein indicated.

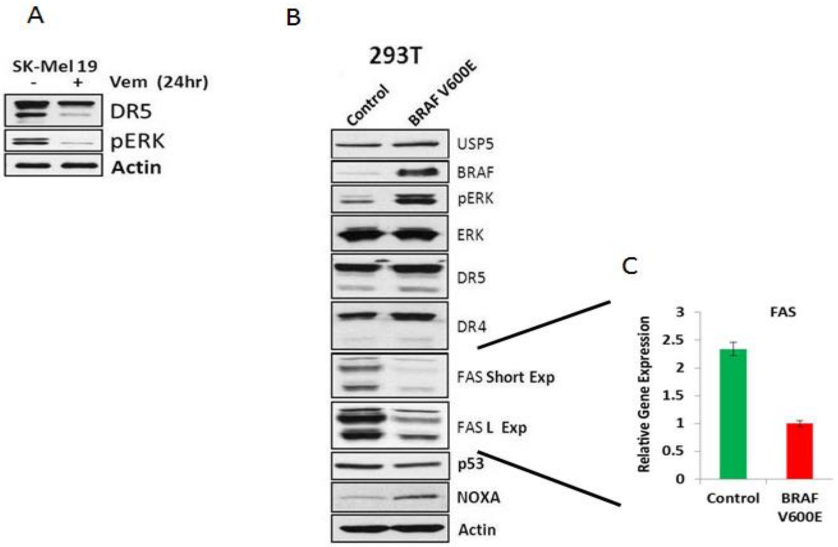

Figure S3: A. SK-Mel19 cells were treated with vehicle alone or 5 µM vemurafenib for 24 hr. Cell lysates were subjected to immunoblotting for the protein indicated. **B.** HEK293T cells were transfected with control or $BRAF^{V600E}$ expression vector. After 48 hr, whole cell lysates were subjected to Usp5, BRAF, pERK, ERK, DR5, DR4, FAS, p53 and NOXA assessment by immunoblotting. Actin was immunoblotted as a protein loading control. **C.** HEK293T cells were subjected to transfection with control or $BRAF^{V600E}$ expression vector and FAS expression was assessed by RT-PCR. The results represent the ave. +/- S.D. of triplicate assays.

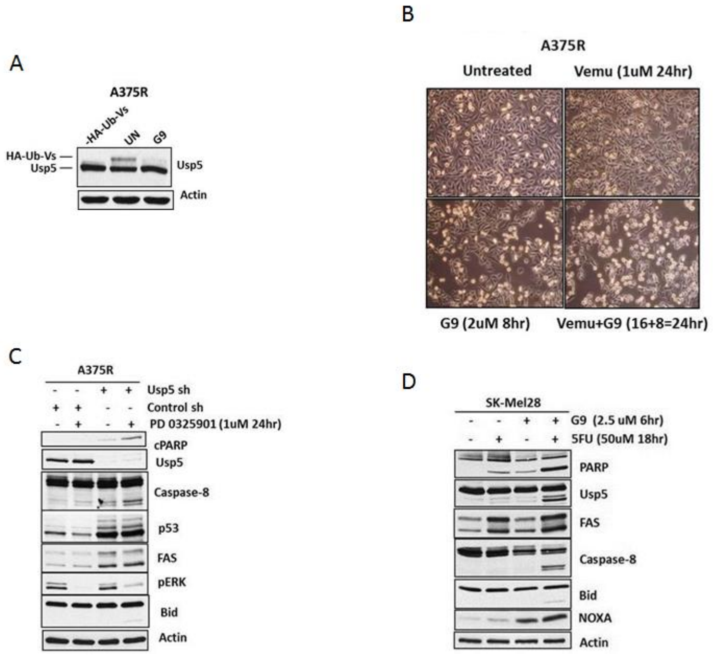

Figure S4: A. A375R cells were incubated with 2.5 µM G9 for 4 hr before Usp5-specific DUB activity was assessed in lysates by HA-UbVS labeling followed by Usp5 blotting. Actin serves as a protein loading control. **B.** A375R cells were treated with vemurafenib or G9 alone or in combination as described. Cell images were acquired using an Olympus FluoView 500 confocal microscope with a 60× water immersion lens and 2× digital zoom (× 120 total magnifications). **C.** Usp5 KD and control A375R cells were treated with PD 0325901 before cell lysates were examined for the protein indicated. **D**. SK-Mel28 cells were treated with 5FU or G9 alone or in combination before cell lysates were examined for the protein indicated.

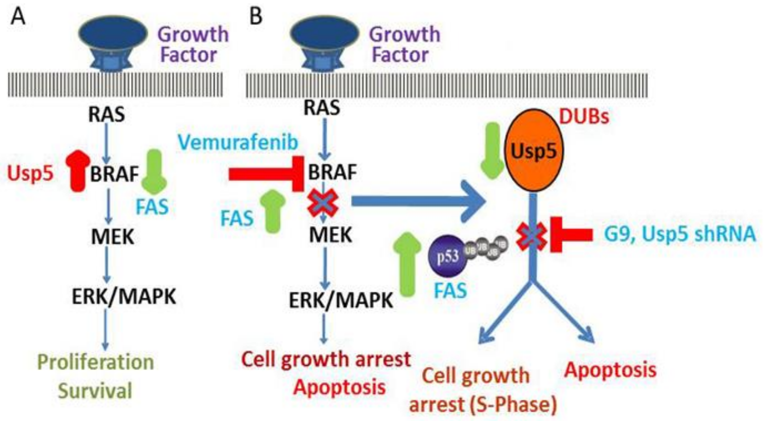

Figure S5: A. BRAF^{V600E} activates Usp5 and promotes cell survival signaling through FAS down-regulation. B. Vemurafenib inhibits mutant BRAF and downstream signaling (ERK/MAPK) while partially suppressing Usp5 activity. These actions activate apoptosis through FAS induction. Usp5 inhibition by G9 or Usp5 shRNA amplifies apoptosis through stabilization of ubiquitinated p53 and induction of FAS.
